# Feeding Parenteral Nutrition in the Neonatal Period Programs Dyslipidemia in Adulthood in Yucatan Miniature Pigs

**DOI:** 10.1016/j.tjnut.2024.08.031

**Published:** 2024-09-11

**Authors:** Raniru S Randunu, Khaled Alawaini, Lee-Anne Huber, Edward W Randell, Janet A Brunton, Robert F Bertolo

**Affiliations:** 1 Department of Biochemistry, Memorial University of Newfoundland, St. John’s, NL, Canada; 2 Department of Animal Biosciences, University of Guelph, Guelph, ON, Canada; 3 Discipline of Laboratory Medicine, Faculty of Medicine, Memorial University of Newfoundland, St. John’s, NL, Canada

**Keywords:** nutritional programming, dyslipidemia, IUGR, methyl nutrients, pigs, TPN

## Abstract

**Background:**

Early nutritional challenges can lead to permanent metabolic changes, increasing risk of developing chronic diseases later in life. Total parenteral nutrition (TPN) is a life-saving nutrition regimen, used especially in intrauterine growth-restricted (IUGR) neonates. Early TPN feeding alters metabolism, but whether these alterations are permanent is unclear. Programmed metabolism is likely caused by epigenetic changes due to imbalances of methyl nutrients.

**Objectives:**

We sought to determine whether feeding TPN in early life would increase risk of developing dyslipidemia in adulthood and whether supplementing the methyl nutrients betaine and creatine to TPN would prevent this development. We also sought to determine whether IUGR exacerbates the effects of neonatal TPN on lipid metabolism in adulthood.

**Methods:**

Female piglets (*n* = 32; 7 d old) were used in 4 treatments: 24 normal-weight piglets were randomly assigned to sow-fed (SowFed), standard TPN (TPN-control), and TPN with betaine and creatine (TPN-B+C); 8 IUGR piglets were fed control TPN (TPN-IUGR) as a fourth group. After 2 wk of treatment, all pigs were then fed a standard solid diet. At 8 mo old, central venous catheters were implanted to conduct postprandial fat tolerance tests.

**Results:**

Feeding TPN in the neonatal period led to dyslipidemia in adulthood, as indicated by higher postprandial triglyceride (TG) levels in TPN-control (*P* < 0.05), compared with SowFed. IUGR piglets were particularly sensitive to neonatal TPN feeding, as TPN-IUGR piglets developed obesity and dyslipidemia in adulthood, as indicated by greater backfat thickness (*P* < 0.05), higher liver TG (*P* < 0.05), slower postprandial TG clearance (*P* < 0.05), and elevated fasting plasma nonhigh-density lipoprotein-cholesterol (*P* < 0.01), and nonesterified fatty acids (*P* < 0.001), compared with TPN-control.

**Conclusions:**

Feeding TPN in early life increases the risk of developing dyslipidemia in adulthood, especially in IUGR neonates; however, methyl nutrient supplementation to TPN did not prevent TPN-induced changes in lipid metabolism.

## Introduction

Dyslipidemia is an important metabolic predictor of many chronic diseases, such as cardiovascular diseases (CVD), obesity, insulin resistance, and fatty liver [1]. Recent evidence has shown that nutritional or environmental insults during early life (pre- and postnatal) can program metabolism via epigenetics and increase the risk for these chronic diseases in later life [2]. Total parenteral nutrition (TPN) is a nutritional intervention often given during the neonatal period to increase survival rates of neonates when they are born premature, intrauterine growth-restricted (IUGR), or when the enteral feeding is not compatible due to pathological conditions [3,4]. Feeding TPN to neonates leads to gut atrophy and altered metabolism [5], resulting in insulin resistance, fatty liver, and acute oxidative damage to organs [[6], [7], [8], [9]]. These metabolic consequences are associated with dyslipidemia, leading to CVD and metabolic syndrome [10,11]. Importantly, TPN administered in early life, during an epigenetic programming window, can exert profound metabolic stress on the neonate during a particularly sensitive stage of development [6,7]. However, it is unknown whether this TPN-induced metabolic dysfunction persists beyond adolescence into adulthood.

Studies have also shown that lower birth weight is linked with poorer health outcomes later in life [12,13]. We have recently demonstrated in pigs that IUGR neonates develop a higher risk for chronic diseases in adulthood [14,15]. Rapid catch-up growth during postnatal and early childhood are important in predicting future health problems such as dyslipidemia, which can predispose an individual to develop metabolic syndrome and CVD later in life [16,17]. TPN feeding has become an essential part of the medical management of preterm and IUGR infants, such that ∼90% of infants <1500 g and ∼80% of infants who fail to establish enteral feeds by days 3–5 are prescribed TPN [18]. Therefore, IUGR infants are particularly vulnerable to TPN-induced metabolic changes that can become lifelong [19].

Studies in animal models of early programming of adult disease suggest that dysregulation of methyl metabolism may explain programming that leads to adult chronic diseases by permanently altering epigenetic patterns of key genes [20,21]. Indeed, methylation patterns can be altered by perinatal dietary methyl supply [22,23] and some of these epigenetic changes are reversible with changes in dietary methyl supply [24]. However, it is unknown if changes in methyl supply and demand in early life can permanently alter epigenetics. Methionine is the primary source of methyl groups, and most transmethylation reactions occur in the liver, although the gut is also responsible for a significant proportion of methionine metabolism [25,26]. Moreover, IUGR has also been shown to reduce hepatic methionine metabolism capacity [27]. Importantly, both the gut and liver often become dysfunctional during prolonged TPN feeding, reducing methionine and methyl metabolic capacity and potentially limiting methyl availability for epigenetic processes [28,29].

In total, the various transmethylation reactions consume a significant proportion of methionine flux; in particular, synthesis of creatine can consume ∼35% of dietary methionine in neonatal piglets [30], and dietary provision of creatine can spare methionine for other reactions [31]. We have recently shown that if 1 methylation pathway is enhanced (e.g., creatine synthesis), it diminishes the partitioning of the limited methyl groups among the remaining methylation reactions, especially phosphatidylcholine (PC) synthesis, which can lead to fatty liver development [32]. Indeed, creatine supplementation has been shown to prevent fatty liver [33]. Similarly, supplementing betaine, a methyl donor that can replenish methionine availability, was also shown to prevent fatty liver and reduce plasma homocysteine levels via increased remethylation [33]. Hence, betaine and creatine (B+C) can increase methyl group availability, although their role in epigenetic programming is unclear. Neither of these nutrients is currently provided in commercial TPN products.

The objectives of this study were to investigate: *1*) whether the metabolic effects of TPN feeding in the neonatal period persist into adulthood; *2*) if the addition of betaine and creatine to TPN prevents the deleterious effects of TPN on lipid metabolism in adulthood; and *3*) whether IUGR will exacerbate the effects of neonatal TPN on lipid metabolism in adulthood. We used the pig as our animal model as it is the most relevant species for modeling human lipid metabolism and has been established as a model for TPN and for investigating early programming effects on risk for adult diseases [15,34].

## Materials and Methods

### Animals and surgical catheter implantation

Animal care and handling procedures were approved by the Memorial University of Newfoundland Animal Care Committee in accordance with Canadian Council on Animal Care guidelines. We utilized only female pigs in this study because females are more prone than males to develop early biomarkers of metabolic syndrome [14,35]. Suckling female Yucatan miniature piglets (*n =* 32; 7 d old) were obtained from the Memorial University of Newfoundland breeding colony and used in 4 treatments: normal birthweight piglets fed standard TPN (TPN-control); normal birthweight piglets fed TPN supplemented with betaine and creatine (TPN-B+C); normal birthweight suckling piglets (SowFed); and IUGR piglets fed standard TPN (TPN-IUGR). These 32 piglets were taken from 13 litters, with 8 piglets assigned to each group. IUGR was defined as ∼65% of the birth weight of the largest littermate [36]. At ∼7 d old, all piglets underwent surgical procedures to implant 2 central venous catheters [37] (study day 0). TPN-fed piglets were housed for 14 d in individual metabolic cages fitted with a swivel and tether system (Lomir Biomedical) that allowed free movement while facilitating continuous intravenous diet infusion into a central vein catheter. Room temperature was maintained for piglets at ∼27°C and supplemental heat was provided via heat lamps; ambient lights provided 12-h light-dark cycles. SowFed piglets were returned to the sow after surgical recovery and allowed to suckle until study day 14 (all piglets resumed suckling within 2–3 h of being returned to the sow). After 14 d of treatment, piglets were anesthetized as above, and catheters were removed. After recovery, all piglets were acclimated to a standard grower diet, which was fed until the end of the study. Groups of 4 pigs were housed together for 8 mo and fed 2% of their cumulative body weights as a group, adjusted every 2 wk after body weights were measured. Because Yucatan miniature pigs tend to become obese, we fed diets at 2% of body weight during the grow-out phase, which is sufficient for normal growth and maintenance of Yucatan miniature pigs, as per our herd maintenance guidelines. Water was available 24 h ad libitum and a 12-h day-night cycle was maintained (lights on 800–2000 h).

At 8 mo of age, 2 venous catheters (inner diameter, 1.0 mm; outer diameter, 1.8 mm; Tygon Medical Tubing, Saint Gobain Performance Plastic Corp.) were implanted in the femoral vein [38] for blood sampling during the oral fat tolerance test (OFTT). Briefly, anesthesia was induced with a dexmedetomidine, azepromazine, and alfaxalone mixture, and maintained with 1.5% isoflurane. Two blood sampling catheters were then inserted into the left femoral vein, tunneled under the skin, and exteriorized between the shoulder blades. After the surgery, 300 μg intravenous buprenorphine hydrochloride (Temgesic; Schering-Plough Ltd.), and intravenous trimethoprim 2.8 mg⋅kg body weight^–1^ and sulfadoxine (1.4 mg⋅kg body weight^–1^) (Borgal; Intervet Ltd.) were given for 3 d postoperatively and each pig was housed individually for the remainder of the study, including 8–10 d for postsurgical recovery [15].

### Diets

The TPN diets (Table 1) were prepared according to Dodge et al. [37] under aseptic conditions. The complete TPN diet provided 1.1 MJ of metabolizable energy·kg body weight^–1^·d^–1^ with glucose (24.5 g·kg body weight^−1^·d^−1^), lipid (9.3 g·kg body weight^–1^·d^–1^ as 20% SMOFlipid, Fresenius Kabi), and protein (15 g·kg body weight^–1^·d^–1^ as free amino acids) supplying 35%, 40%, and 25% of energy. The amino acid composition is shown in Table 1. TPN was provided to the TPN groups at 12 mL·kg body weight^–1^·h^–1^. All vitamins and macro minerals were supplied at >100% of the estimated requirements for neonatal piglets [37]. Just prior to infusion, multivitamins (Multi12/K1 Pediatric, Baxter Corporation), iron dextran (Bimeda-MTC Animal Health), trace elements (Sigma-Aldrich Canada), and SMOFlipid emulsion were added to each diet bag. Because SMOFlipid 20% emulsion (mixture of soybean, medium-chain triglycerides [TGs], olive oil, and fish oil) is becoming the standard of care for many neonates, we decided to use this emulsion instead of Intralipid. SMOFlipid was administrated at a rate of 1.9 mL·kg body weight^–1^·h^–1^. For the TPN-B+C group, TPN was supplemented with betaine (235 mg·kg body weight^–1^·d^–1^) and creatine (118 mg·kg body weight^–1^·d^–1^). Betaine was supplied at a molar equivalent of the piglet methionine requirement [39], and creatine was supplemented to match the piglet creatine accretion rate [30]. Diet bags were weighed routinely to record the amount of diet infused. For the SowFed piglets, intakes were not measured, but were estimated from literature data. Milk consumption of 7-d-old piglets is ∼350 mL·kg body weight^–1^·d^–1^ [30]. The macronutrient composition of sow milk at 7 d old is 5.4% total protein, 7.6% fat, and 5.2% lactose [40]. Therefore, the macronutrient intake of suckling pigs was estimated as 19% lactose, 62% fat, and 19% protein. After the dietary treatment period of 14 d (i.e., at 21 d of age), each piglet was acclimated with milk replacer (Grober Nutrition Inc.) for 2–3 d ad libitum and weaned onto a standard pelleted grower pig diet (Table 2; Eastern Farmers Co-op). This grower diet delivered 67% of energy as carbohydrate, 12% as fat, and 21% as protein.

**TABLE 1:** Amino acid profile of TPN diets

	TPN-control diet (g⋅L^−1^)	TPN betaine + creatine diet (g⋅L^−1^)
Alanine	5.89	5.89
Arginine	3.65	3.65
Aspartic acid	3.32	3.32
Cysteine	0.76	0.76
Glutamic acid	5.72	5.72
Glycine	1.47	1.47
Histidine	1.69	1.69
Isoleucine	2.51	2.51
Leucine	5.67	5.67
Lysine hydrochloride	5.58	5.58
Methionine	1.04	1.04
Phenylalanine	3	3
Proline	4.52	4.52
Serine	3.11	3.11
Taurine	0.27	0.27
Threonine	2.23	2.23
Tryptophan	1.14	1.14
Tyrosine	0.44	0.44
Valine	2.89	2.89
Betaine hydrochloride	0	1.29
Creatine monohydrate	0	0.57

Abbreviation: TPN, total parenteral nutrition.

**TABLE 2:** Composition of the grower diet (12.1 MJ digestible energy⋅kg^−1^ and 154 g protein⋅kg^−1^)

Energy (% total energy)	
Complex carbohydrate	67
Fat	12
Protein	21
Ingredients (g⋅kg^–1^ dry matter)	
Wheat shorts	400.5
Canola	49.0
Meat meal	19.0
Limestone	13.0
Corn gluten feed	40.0
Ground barley	297.0
Oats	175.0
Vitamin mix	0.8
Mineral mix	1.0
Sodium chloride	4.7

### Body measurements

Body weight was measured every day during the experimental dietary period (i.e., 7–21 d of age), and body weight was measured every 2 wk throughout the grow-out phase of the study (i.e., from 1 to 9 mo). Feed conversion ratio (FCR) was calculated by dividing the predicted feed intake of each pig (2% of their body weight) by the body weight they gained in each phase (1–4 mo/4–6 mo/6–8 mo). Because sexual maturity occurs in Yucatan miniature pigs around 4 mo, we divided the growth data by development 1–4 mo (presexual maturity), 4–6 mo (peri-sexual maturity), and 6–8 mo (postsexual maturity) (Table 3) [14]. Fractional growth rate (FGR) was calculated by dividing body weight gain for a given period of time by the initial body weight of that time frame.

**TABLE 3:** Summary of body weight, growth rate, FGR, FCR, and body measurements in TPN-control, TPN-B+C, TPN-IUGR, and SowFed Yucatan miniature pigs

Developmental period	Age	TPN-control	TPN-B+C	TPN-IUGR	SowFed
Body weight (kg)					
Birth	1 d	0.98 ± 0.17	1.02 ± 0.12	0.67 ± 0.10^1^	1.01 ± 0.15
Neonate – TPN start	7 d	1.74 ± 0.18	1.734 ± 0.24	1.25 ± 0.16^2^	1.80 ± 0.35
Neonate – TPN end	21 d	3.10 ± 0.20	3.15 ± 0.24	2.44 ± 0.21^3^	3.53 ± 0.88
Neonate – weaning	1 mo	3.54 ± 0.88	3.77 ± 0.61	3.57 ± 1.08	4.81 ± 1.87
Sexual maturity	4 mo	14.99 ± 3.29	15.83 ± 2.77	17.46 ± 2.54	18.06 ± 2.86
Postsexual maturity	8 mo	35.05 ± 7.62	34.43 ± 5.49	41.74 ± 4.63	37.87 ± 7.49
Body weight growth rate (g⋅d^−1^)					
Neonate	7–21 d	97.1 ± 6.2	101.1 ± 7.5	83.0 ± 8.5	117.9 ± 48.7
Presexual maturity	1–4 mo	130.1 ± 38.5	135.6 ± 30.9	171.8 ± 25.1^3^	151.8 ± 31.9
Sexual maturity	4–6 mo	143.7 ± 52.5	148.5 ± 45.4	203.7 ± 28.7^3^	165.1 ± 57.1
Postsexual maturity	6–8 mo	190.2 ± 39.9	169.0 ± 34.9	194.7 ± 51.0	164.0 ± 59.3
Fractional growth rate (g⋅kg body weight^−1^⋅d^−1^)					
Neonate	7 d–21 d	40.1 ± 3.1	41.6 ± 5.1	47.6 ± 4.3	45.4 ± 11.7
Presexual maturity	1–4 mo	13.9 ± 2.7	13.7 ± 2.1	15.2 ± 3.3	13.0 ± 2.6
Sexual maturity	4–6 mo	7.3 ± 1.6	7.3 ± 1.7	8.5 ± 1.3	6.4 ± 2.6
Postsexual maturity	6–8 mo	6.1 ± 0.6	5.3 ± 0.6	5.6 ± 1.2	5.0 ± 1.1
Average feed conversion ratio					
Presexual maturity	1–4 mo	1.6 ± 0.2	1.6 ± 0.1	1.5 ± 0.3	1.7 ± 0.2
Sexual maturity	4–6 mo	3.3 ± 1.0	3.4 ± 1.1	2.6 ± 0.5	3.4 ± 1.1
Postsexual maturity	6–8 mo	3.8 ± 0.6	4.5 ± 1.2	4.5 ± 1.3	5.4 ± 2.1
Body measurements					
Crown to rump length (cm)	9 mo	112.1 ± 6.1	111.8 ± 6.3	118.1 ± 3.2	116.3 ± 6.9
Abdominal circumference (cm)	9 mo	84.7 ± 6.8	82.8 ± 6.8	88.3 ± 4.1	84.4 ± 7.2
Chest girth (cm)	9 mo	79.4 ± 4.3	79.3 ± 3.7	82.9 ± 4.3	77.7 ± 4.0
Subcutaneous fat thickness (mm)	9 mo	43.2 ± 5.5	43.7 ± 3.8	50.0 ± 5.0^3^	45.1 ± 5.6

Values are means ± SD; *n* = 7–8.

Abbreviations: FCR, feed conversion ratio; FGR, fractional growth rate; TPN, total parenteral nutrition; TPN-control, TPN-control diet; TPN-B+C, TPN-control diet supplemented with betaine and creatine; TPN-IUGR, IUGR piglets fed TPN-control diet; SowFed, suckled.

1 *P* < 0.0001; one-way analysis of variance with Dunnett’s post hoc test comparing with TPN-control.

2 *P* < 0.001; one-way analysis of variance with Dunnett’s post hoc test comparing with TPN-control.

3 *P* < 0.05; one-way analysis of variance with Dunnett’s post hoc test comparing with TPN-control.

### Oral fat tolerance test

After 8–10 d of surgery recovery at 8 mo old, adult pigs were fasted for 18 h prior to the OFTT, and on the test day, a baseline blood sample [10 mL in EDTA tubes (Becton, Dickinson and Company)] was collected via the venous catheter. Immediately after, pigs were presented with a high-fat meal bolus (1.5 g fat⋅kg body weight^–1^), which was made with margarine (25%) (Central Dairies) and lard (75%) (Loblaw Inc.). The fat bolus was blended with some ground grower pig diet (∼5% of the total bolus) (Eastern Farmers Co-op) to enhance the absorption of fatty acids in the presence of some carbohydrates and protein [41] and to improve palatability. After the meal was presented to the pigs, they were allowed to eat for 1 h to consume the entire bolus, and water was provided ad libitum during the test. Starting from the bolus introduction, blood samples (10 mL), were collected hourly for the next 11 h. Half of each blood sample was immediately centrifuged at 4000 × *g* for 15 min at 4°C to separate plasma, frozen at –80°C and the other half was centrifuged at 15,500 × *g* for 20 min at 12°C for separation of chylomicron (CM) and chylomicron-free (CMF) fractions [15,42]. All plasma, CM, and CMF fractions were analyzed for TG concentrations using a commercially available enzymatic assay kit (Sekisui Diagnostics PEI Ltd.). Total AUC (calculated using the trapezoid method from baseline to final TG measurements for each plasma and CM fraction), peak TG concentration, peak TG concentration adjusted to the baseline TG, and time to peak TG were used to quantify the total plasma and CM TG responses during the OFTT [15].

### Necropsy

After the OFTT (∼1 mo), the pigs were anesthetized with 105 mg⋅kg body weight^−1^ of sodium pentobarbital (Euthanyl, Biomeda-MTC Cambridge) and mechanically ventilated until the organs were removed to prevent tissue death from hypoxia; pigs died from exsanguination after organs were removed. Blood samples were collected into EDTA tubes by cardiac puncture and assayed for lipids, as described ahead. Organs were removed and weighed, and samples were flash-frozen using a freeze clamp and liquid nitrogen and stored at –80°C until further analyses. Back fat thickness, a measure of subcutaneous fat, was measured with a ruler on the carcass at the dorsal midline, immediately caudal to the last rib. Crown to rump length, abdominal circumference (at the umbilicus), and chest girth (at sternum) were also measured at necropsy.

### Plasma lipid, glucose, and insulin analyses and CETP activity

Blood samples collected at necropsy and fasting monthly blood samples were centrifuged immediately at 4000 × *g* for 15 min to separate plasma, and fresh plasma was assayed for concentrations of plasma total cholesterol (TC), TG, high-density lipoprotein (HDL)-cholesterol, and free cholesterol using enzymatic assay kits (Sekisui Diagnostics PEI Ltd.). Plasma non-HDL-cholesterol was calculated by subtracting values of HDL-cholesterol from TC. Plasma nonesterified fatty acid (NEFA) was assayed using an enzymatic assay kit (Fujifilm Wako Diagnostics USA Corporation). Plasma glucose concentrations were measured using an enzymatic assay kit (Sigma-Aldrich) and plasma insulin concentrations were measured using the Human Insulin ELISA kit (ab100578 Human Insulin ELISA kit; Abcam Inc.) following assay procedures. Lipoprotein fractions were collected by sequential density ultracentrifugation of fresh necropsy plasma [43]. Necropsy plasma was centrifuged at 15,500 × *g* for 20 min at 12°C to separate CM and the infranatant was subjected to sequential density ultracentrifugation to collect lipoprotein fractions, VLDL, LDL, and HDL. TC and TG levels were measured in these fractions using enzymatic assay kits. Plasma cholesterol ester transfer protein (CETP) activity was measured using a CETP Activity Assay Kit (ab196995 CETP Activity Assay Kit II; Abcam Inc.) following assay instructions.

### Liver TG analysis and tissue lipoprotein lipase activity

Lipids were extracted from liver tissue (left lateral lobe) using the chloroform:methanol (2:1, vol/vol) extraction technique [44], and the lipid-containing phase was evaporated to yield dried lipids. Dried lipids were then dissolved using isopropanol and analyzed for TG and TC by enzymatic assay kits. Quantitative lipoprotein lipase (LPL) was measured in subcutaneous adipose tissue, gastrocnemius muscle, and cardiac tissue (left ventricle) using an LPL activity assay kit (Abcam Inc.), following assay guidelines.

### Gut parameters and histology

Different segments of the gut (duodenum, proximal jejunum, mid jejunum, and ileum) were excised, emptied, and collected during necropsy. First, the duodenum (from the pyloric sphincter to the duodenojejunal flexure) was removed, emptied, and measured for length and weight, followed by the remaining small intestine. The first 30 cm of the remaining small intestine was considered as the proximal jejunum, 30 cm from the midpoint of the small intestine was considered as mid jejunum, and the terminal 30 cm of the small intestine was considered as the ileum. Sections (∼1 cm) from each intestinal segment were placed in neutral-buffered 10% formalin (Sigma Chemical) for histological analysis. The data were expressed as small intestinal weight per length (mg⋅cm^–1^) for all 4 segments. Formalin-fixed intestinal sections were processed, and 10 measurements of villus height and crypt depths per sample were measured by the same individual in a blinded fashion, as described elsewhere [37].

### Plasma and tissue metabolites

Concentrations of plasma betaine, choline, and dimethylglycine (DMG) levels were quantified by HPLC (Waters Alliance 2795, Waters Corporation; Atlantis HILIC Silica 3 μm 2.1 × 100-mm column) with a tandem mass spectrometer (MS) (Micromass Ultima Triple-Quad MS), as previously described [45,46]. Multiple-reaction monitoring mode was used to detect the compounds with the following transitions: [^2^H_11_] betaine 129➔68, betaine 118➔59, [^2^H_9_ methyl] choline 113➔69, choline 104➔60, and DMG 104➔58. Plasma concentrations were calculated using calibration standards made using dialyzed plasma spiked with betaine, choline, and DMG. [^2^H_9_-methyl] choline and [^2^H_11_] betaine were used as the internal standards [45]. Final concentrations of betaine, choline, and DMG were calculated using MassLynx Software (Waters Corporation).

Plasma total homocysteine, cysteine, and glutathione concentrations were determined using reverse-phase HPLC and fluorescence detection of ammonium 7-fluoro 2-oxa-1,3-diazole-4- sulfonate thiol adducts [47]. Liver homocysteine and cysteine concentrations were measured using the same method with modifications using 2-mercapto propionyl glycine as the internal standard [48]. Plasma methionine concentrations were measured using reverse-phase HPLC after derivatization with phenyl isothiocyanate [49].

After lipids were extracted from the liver [44], PC and phosphatidylethanolamine (PE) were isolated using thin-layer chromatography, as previously described [48]. The quantification of liver PC and PE was then measured by measuring total phosphate using a modified Bartlett method [50] described elsewhere [48].

### RNA extraction and real-time quantitative polymerase chain reaction

Total RNA was extracted from liver samples using the Trizol method [51]. Genomic DNA contamination in RNA samples was eliminated using DNAse enzyme (cat. # M610A, Promega). The concentration of the extracted RNA samples from the liver was determined using NanoDrop 2000 (Thermo Fisher Scientific). Confirmation of the integrity of each RNA sample was then determined using 1.2% agarose gel. Complementary DNA (was synthesized from the extracted RNA samples using reverse transcription (cat # A3500, Promega). Real-time quantitative polymerase chain reaction (qPCR) primers for microsomal triglyceride transfer protein (MTTP) [52], β-actin [53], and glyceraldehyde 3-phosphate (GAPDH) [54] (Integrated DNA Technologies) were verified using NCBI primer blast (www.ncbi.nlm.nih.gov/tools/primer-blast/). The forward and reverse sequence for each primer pairs were MTTP forward – GCCAGGTCTTCCAGAGCGAGTG; MTTP reverse-TGCCGTCCTGAGGTGCTGAATG; β-actin forward-CAC GCC ATC CTG CGT CTG GA; β-actin reverse-AGC ACC GTG TTG GCG TAG AG); GAPDH forward-ATCCTGGGCTACACTGAGGA; and GAPDH reverse-TGTCGTACCAGGAAATGAGCT. GenBank accession numbers for the primers were NM_214185 for MTTP, DQ845171 for β-actin, and NM_001206359.1 for GAPDH. The qPCR amplification was initiated using SYBR Green Supermix (cat # 1708882, Bio-Rad), and the samples were run using the Mastercycler ep realplex system (Eppendorf). The delta Ct values were calculated for the MTTP and reference genes. Both β-actin and GAPDH were used to normalize the expression levels of the MTTP gene, accounting for their primer efficiencies [55].

### Statistical analyses

The experimental groups were compared using one-way analysis of variance and the differences among groups were determined using Dunnett’s post hoc test (GraphPad Prism 8.0; GraphPad Software), with TPN-control assigned as the control group. We aimed to compare the long-term effects of early TPN support (TPN-control); thus, we compared the TPN-control group with the SowFed group, which served as the clinical control. Other aims of this study were to compare early TPN support (TPN-control) to TPN supplemented with betaine and creatine (TPN-B+C) and to IUGR neonates fed TPN (TPN-IUGR). The results are reported as mean ± SD. Pearson’s correlation was used to compare the relationship between TG in VLDL and the plasma TG clearance rate. Differences were considered statistically significant if *P* < 0.05.

## Results

### Growth

The growth data were partitioned into 4 developmental phases, with reference to sexual maturity, which in Yucatan miniature pigs occurs between 4 and 6 mo of age. The lower body weight of the TPN-IUGR piglets persisted throughout the TPN phase to 21 d old (Table 3). SowFed and TPN-B+C piglets grew no differently from TPN-control piglets during the TPN feeding phase. By 1 mo old, body weights of TPN-IUGR piglets caught up to TPN-control and there was no difference in body weight compared with TPN-control group beyond 1 mo. The body weight growth rates were greater for TPN-IUGR pigs between 1–4 and 4–6 mo developmental stages compared with the TPN-control pigs, but there were no differences in the 6–8 mo phase among the groups. Although body weights were not different between groups from weaning to sexual maturity, body growth rates were higher in TPN-fed IUGR pigs, possibly due to their programmed metabolism leading to catch-up growth. FGR, average FCR, and body measurements at 9 mo were not different compared with the TPN-control group (Table 3). Subcutaneous fat thickness was greater in TPN-IUGR pigs only; other body measurements were not different.

### Oral fat tolerance test

**FIGURE 1:**
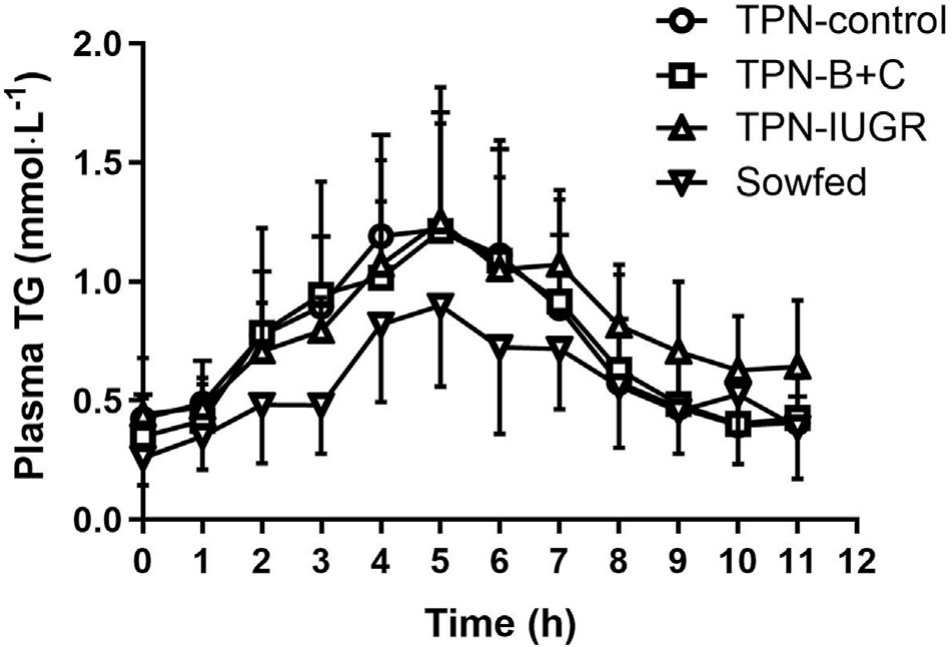
Plasma TG concentrations in TPN-control, TPN-B+C, TPN-IUGR, and SowFed Yucatan miniature pigs before and after administration of a high-fat bolus. Values are mean ± SD; *n* = 7–8. The fat bolus was given at time 0 h and the pigs were allowed to consume the diet for 1 h. TPN, total parenteral nutrition; TPN-control, TPN-control diet; TPN-B+C, TPN-control diet supplemented with betaine and creatine; TPN-IUGR, IUGR piglets fed TPN-control diet; SowFed, suckled.

**FIGURE 2:**
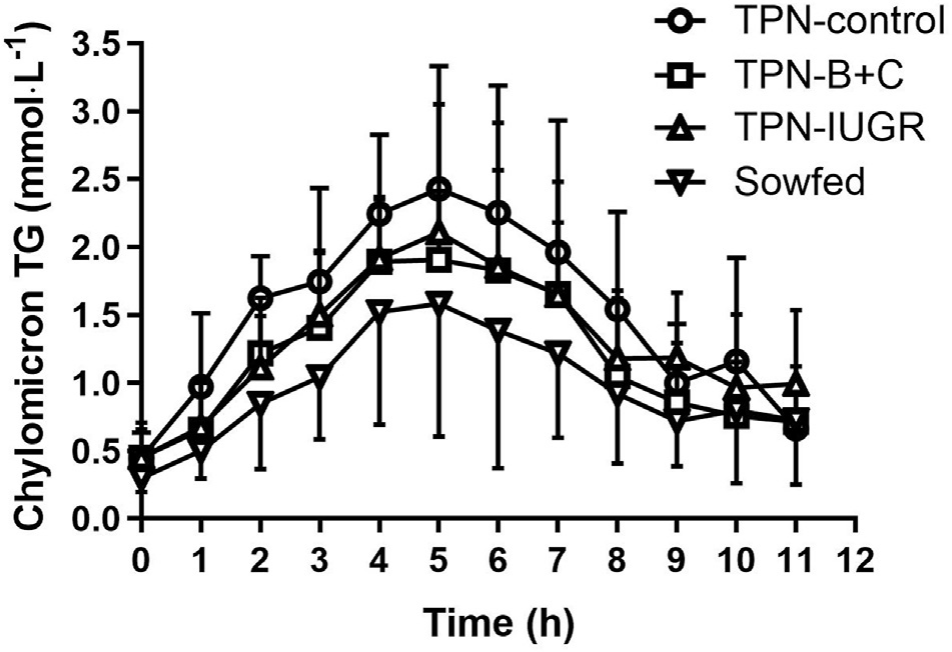
Plasma CM TG concentrations in TPN-control, TPN-B+C, TPN-IUGR, and SowFed Yucatan miniature pigs before and after administration of a high-fat bolus. Values are mean ± SD; *n* = 6–8. The fat bolus was given at time 0 h and the pigs were allowed to consume the diet for 1 h. TPN, total parenteral nutrition; TPN-control, TPN-control diet; TPN-B+C, TPN-control diet supplemented with betaine and creatine; TPN-IUGR, IUGR piglets fed TPN-control diet; SowFed, suckled.

**FIGURE. 3:**
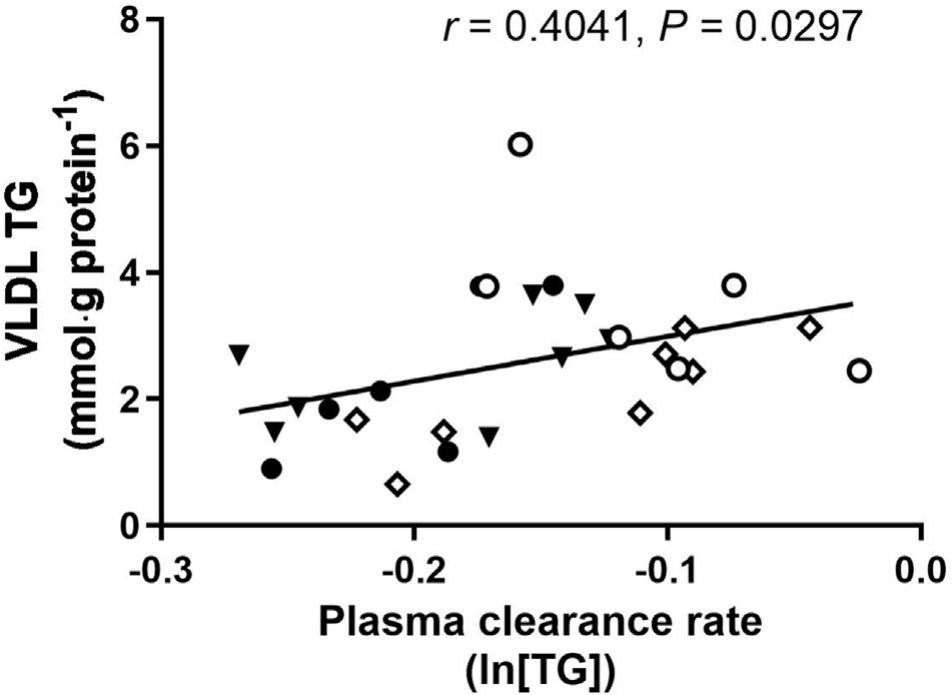
Correlation between TG concentration in VLDL fractions of fasted plasma at 9 mo (mmol⋅g protein^–1^) and plasma TG clearance rate (ln [TG]) at OFTT. TG concentration in VLDL fractions of fasted plasma (mmol⋅g protein^–1^) at 9 mo correlated positively with plasma TG clearance rate (*r* = 0.4041; *P* = 0.0297); *n* = 6–8. Each symbol represents an individual pig; TPN-control (●); TPN-B+C (▼); TPN-IUGR (○), SowFed (◊). TPN, total parenteral nutrition; TPN-control, TPN-control diet; TPN-B+C, TPN-control diet supplemented with betaine and creatine; TPN-IUGR, IUGR piglets fed TPN-control diet; SowFed, suckled.

After ingesting the bolus of fat, the AUC of plasma TG was lower in the SowFed group than for the TPN-control pigs (Figure 1; Table 4). Further analysis showed that the SowFed group exhibited lower peak TG concentration and adjusted peak TG concentration (Table 4) compared with the TPN-control pigs. However, no differences were observed in the baseline TG concentrations among the groups (Table 4). The rate of TG clearance was slower in the TPN-IUGR group than in the TPN-control group, suggesting delayed postprandial TG clearance in TPN-fed IUGR pigs in adulthood. Similar to TG concentrations in plasma, the AUC of TG concentrations in CM was lower in SowFed pigs than in TPN-control pigs (Figure 2; Table 5). Peak CM TG concentrations and adjusted peak CM TG concentrations were also lower in the SowFed group compared with TPN-control pigs, whereas the baseline CM TG levels show no difference among the groups. TG concentrations in VLDL fractions of fasted plasma were positively correlated with plasma clearance rate during OFTT (Figure 3).

**TABLE 4:** Summary of plasma TG in OFTT performed in TPN-control, TPN-B+C, TPN-IUGR, and SowFed Yucatan miniature pigs at 9 mo

	TPN-control	TPN-B+C	TPN-IUGR	SowFed
Baseline TG (mmol⋅L^−1^)	0.42 ± 0.10	0.35 ± 0.16	0.44 ± 0.24	0.26 ± 0.12
Total peak area (mmol⋅L^−1^⋅h^−1^)	9.67 ± 1.80	8.40 ± 3.09	9.45 ± 2.01	6.20 ± 2.07^1^
Peak TG (mmol⋅L^−1^)	1.60 ± 0.32	1.30 ± 0.39	1.39 ± 0.52	0.97 ± 0.34^1^
Adjusted peak TG (mmol⋅L^−1^)^2^	1.14 ± 0.26	1.03 ± 0.36	1.08 ± 0.36	0.67 ± 0.23^1^
Time to peak TG (h)	5.1 ± 1.1	4.7 ± 1.4	5.4 ± 1.1	5.2 ± 0.9
Rate of TG clearance (ln [TG])	0.19 ± 0.05	0.19 ± 0.06	0.11 ± 0.05^a^	0.13 ± 0.06

Values are means ± SD; *n* = 6–8.

Abbreviations: OFTT, oral fat tolerance test; TG, triglyceride; TPN, Total parenteral nutrition; TPN-control, TPN-control diet; TPN-B+C, TPN-control diet supplemented with betaine and creatine; TPN-IUGR, IUGR piglets fed TPN-control diet; SowFed, suckled.

1 *P* < 0.05; one-way analysis of variance with Dunnett’s post hoc test comparing with TPN-control.

2 Adjusted for baseline TG within pig.

**TABLE 5:** Summary of CM TG in oral fat tolerance test in TPN-control, TPN-B+C, TPN-IUGR, and SowFed Yucatan miniature pigs at 9 mo

	TPN-control	TPN-B+C	TPN-IUGR	SowFed
Baseline TG (mmol⋅L^−1^)	0.45 ± 0.19	0.45 ± 0.26	0.45 ± 0.21	0.30 ± 0.10
Total peak area (mmol⋅L^−1^⋅h^−1^)	17.49 ± 4.05	13.89 ± 4.87	12.82 ± 4.34	10.49 ± 5.24^1^
Peak TG (mmol⋅L^−1^)	2.96 ± 0.60	2.38 ± 0.73	2.09 ± 0.85	1.84 ± 0.89^1^
Adjusted peak TG (mmol⋅L^−1^)^2^	2.50 ± 0.64	1.93 ± 0.69	1.64 ± 0.74	1.54 ± 0.82^1^
Time to peak TG (h)	4.9 ± 0.9	6.0 ± 1.3	4.9 ± 1.0	5.6 ± 1.1
Rate of TG clearance (ln [TG])	0.22 ± 0.08	0.21 ± 0.09	0.16 ± 0.07	0.20 ± 0.07

Values are means ± SD; *n* = 6–8.

Abbreviations: CM, chylomicron; TG, triglyceride; TPN, total parenteral nutrition; TPN-control, TPN-control diet; TPN-B+C, TPN-control diet supplemented with betaine and creatine; TPN-IUGR, IUGR piglets fed TPN-control diet; SowFed, suckled.

1 *P* < 0.05; one-way analysis of variance with Dunnett’s post hoc test comparing with TPN-control.

2 Adjusted for baseline TG within pig.

### Fasting lipid concentrations at necropsy

Fasting plasma non-HDL-cholesterol and plasma NEFA levels were higher in TPN-IUGR pigs than for TPN-control pigs at 9 mo (i.e., necropsy) (Table 6). TPN-fed IUGR pigs had higher liver TG levels than TPN-control pigs at 9 mo (Table 6). Lipoprotein subfraction analysis for lipid parameters at 9 mo showed a lower cholesterol level and higher TG level in VLDL subfraction in TPN-fed IUGR than for TPN-control pigs. A higher cholesterol level in LDL subfraction was also observed in the TPN-IUGR pigs compared with TPN-control pigs (Table 6). Fasting plasma glucose and insulin concentrations were not different in any experimental groups compared with the TPN-control group (Table 6).

**TABLE 6:** Liver, plasma fasting lipid profile, plasma fasting lipoprotein TG, TC, glucose, and insulin profile in Yucatan miniature pigs at 9 mo in TPN-control, TPN-B+C, TPN-IUGR, and SowFed groups

	TPN-control	TPN-B+C	TPN-IUGR	SowFed
Cholesterol				
Liver, total (mmol⋅g protein^−1^)	9.39 ± 2.53	9.39 ± 2.32	11.16 ± 2.54	8.60 ± 4.07
Plasma, total (mmol⋅L^−1^)	1.405 ± 0.268	1.270 ± 0.216	1.617 ± 0.399	1.453 ± 0.117
Plasma, total HDL (mmol⋅L^−1^)	0.974 ± 0.189	0.958 ± 0.191	0.867 ± 0.240	0.938 ± 0.067
Plasma, total non-HDL (mmol⋅L^−1^)	0.460 ± 0.190	0.302 ± 0.095	0.750 ± 0.321^1^	0.530 ± 0.114
Plasma-free cholesterol (mmol⋅L^−1^)	0.608 ± 0.174	0.484 ± 0.249	0.507 ± 0.104	0.536 ± 0.082
VLDL (mmol⋅g protein^−1^)	1.562 ± 0.656	1.427 ± 0.491	0.883 ± 0.263^1^	1.421 ± 0.335
LDL (mmol⋅g protein^−1^)	2.284 ± 0.551	2.390 ± 0.727	4.483 ± 2.062^1^	2.640 ± 0.649
HDL (mmol⋅g protein^−1^)	0.946 ± 0.364	0.957 ± 0.489	1.438 ± 0.496	0.762 ± 0.461
Triglycerides				
Liver, total (mmol⋅g protein^–1^)	3.77 ± 2.94	4.13 ± 2.58	9.60 ± 5.73^1^	4.67 ± 4.37
Plasma, total (mmol⋅L^–1^)	0.397 ± 0.110	0.404 ± 0.113	0.422 ± 0.147	0.358 ± 0.106
VLDL (mmol⋅g protein^–1^)	2.125 ± 1.289	2.522 ± 0.863	3.492 ± 1.208^1^	2.127 ± 0.873
LDL (mmol⋅g protein^–1^)	0.364 ± 0.222	0.339 ± 0.148	0.254 ± 0.085	0.307 ± 0.209
HDL (mmol⋅g protein^–1^)	0.025 ± 0.015	0.031 ± 0.018	0.020 ± 0.004	0.018 ± 0.013
Plasma NEFA (mmol⋅L^–1^)	0.09 ± 0.04	0.09 ± 0.04	0.15 ± 0.03^1^	0.08 ± 0.04
Plasma glucose (mmol ⋅L^–1^)	6.7 ± 1.7	7.7 ± 1.1	7.6 ± 1.4	7.6 ± 0.98
Plasma insulin (μU ⋅mL^–1^)	58.3 ± 51.3	55.5 ± 35.0	74.2 ± 52.8	84.3 ± 52.5
Liver				
Phosphatidylcholine (PC) (mmol⋅g protein^–1^)	0.66 ± 0.12	0.61 ± 0.12	0.68 ± 0.12	0.68 ± 0.13
Phosphatidylethanolamine (PE) (mmol⋅g protein^–1^)	0.43 ± 0.06	0.43 ± 0.07	0.47 ± 0.03	0.46 ± 0.06
PC/PE	1.53 ± 0.22	1.42 ± 0.16	1.57 ± 0.16	1.55 ± 0.35

Values are means ± SD; *n* = 6–8.

Abbreviations: TPN, total parenteral nutrition; TPN-control, TPN-control diet; TPN-B+C, TPN-control diet supplemented with betaine and creatine; TPN-IUGR, IUGR piglets fed TPN-control diet; SowFed, suckled.

1 *P* < 0.05; one-way analysis of variance with Dunnett’s post hoc test comparing with TPN-control.

### Intestinal tissue weight, villi length, and crypt depth

Because gut atrophy is a common outcome of neonatal TPN support, we measured intestinal villi length and crypt depth to determine whether intestinal atrophy persists into adulthood. Duodenum and proximal jejunum villus length and crypt depth did not differ among groups at 9 mo (i.e., necropsy) (Table 7). Unit weight of duodenum was higher in SowFed pigs compared with TPN-control pigs, but no differences were observed for other sections of intestine (Table 8).

**TABLE 7:** Summary of duodenal and proximal jejunum villus height and crypt depth in TPN-control, TPN-B+C, TPN-IUGR, and SowFed Yucatan miniature pigs at 9 mo

	TPN-control	TPN-B+C	TPN-IUGR	SowFed
Duodenum villus height (μm)	421 ± 65	353 ± 36	422 ± 72	383 ± 74
Duodenum crypt depth (μm)	249 ± 26	293 ± 84	294 ± 81	273 ± 88
Proximal jejunum villus height (μm)	498 ± 58	508 ± 60	502 ± 71	551 ± 92
Proximal jejunum crypt depth (μm)	235 ± 36	241 ± 48	292 ± 46	277 ± 75

Values are means ± SD; *n* = 6–8. Abbreviations: TPN, total parenteral nutrition; TPN-control, TPN-control diet; TPN-B+C, TPN-control diet supplemented with betaine and creatine; TPN-IUGR, IUGR piglets fed with TPN-control diet; SowFed, suckled.

One-way analysis of variance with Dunnett’s post hoc test comparing with TPN-control: *P* > 0.05.

**TABLE 8:** Summary of intestinal total tissue weight in TPN-control, TPN-B+C, TPN-IUGR, and SowFed Yucatan miniature pigs at 9 mo

	TPN-control	TPN-B+C	TPN-IUGR	SowFed
Duodenum (mg⋅cm^–1^)	651 ± 228	792 ± 183	812 ± 519	957 ± 196^1^
Proximal jejunum (mg⋅cm^–1^)	437 ± 72	450 ± 105	462 ± 49	436 ± 67
Mid jejunum (mg⋅cm^–1^)	400 ± 82	377 ± 56	443 ± 74	468 ± 104
Ileum (mg⋅cm^–1^)	470 ± 136	547 ± 135	562 ± 146	468 ± 145

Values are means ± SD; *n* = 6–8. Abbreviations: TPN, total parenteral nutrition; TPN-control, TPN-control diet; TPN-B+C, TPN-control diet supplemented with betaine and creatine; TPN-IUGR, IUGR piglets fed TPN-control diet; SowFed, suckled.

1 *P* < 0.05; one-way analysis of variance with Dunnett’s post hoc test comparing with TPN-control.

### Enzyme measurements

**FIGURE 4:**
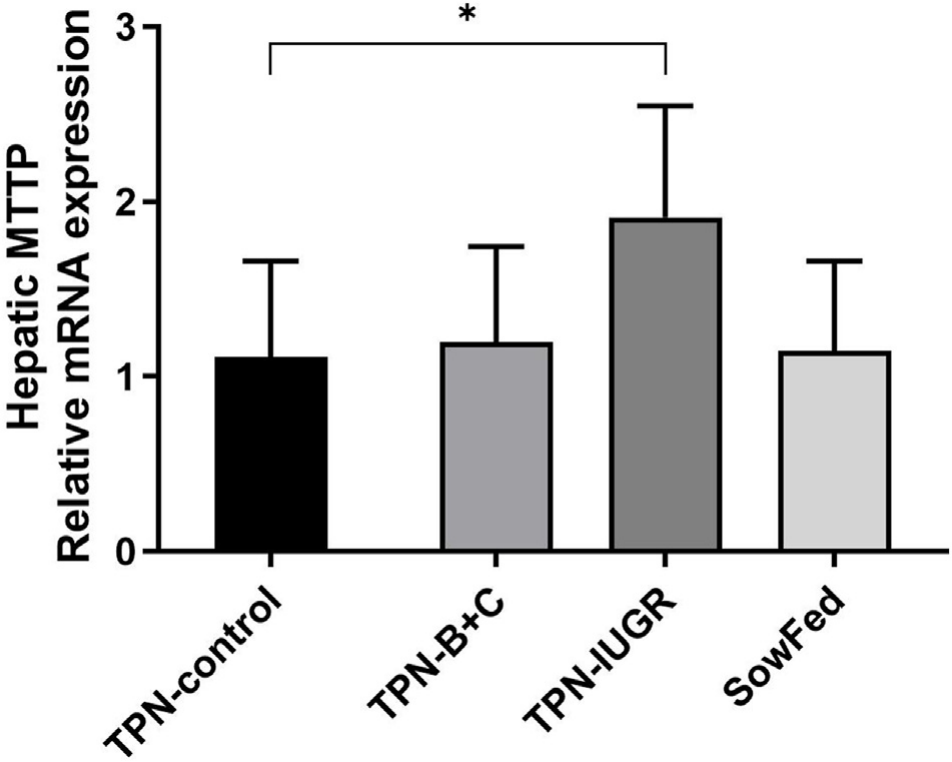
Effects of experimental diets in early life on relative mRNA expression of liver MTTP in TPN-control, TPN-B+C, TPN-IUGR, and SowFed Yucatan miniature pigs at 9 mo. Values are means ± SD; *n* = 7–8. ∗*P* < 0.05; one-way analysis of variance with Dunnett’s post hoc test comparing with TPN-control. MTTP, microsomal triglyceride transfer protein; TPN, total parenteral nutrition; TPN-control, TPN-control diet; TPN-B+C, TPN-control diet supplemented with betaine and creatine; TPN-IUGR, IUGR piglets fed TPN-control diet; SowFed, suckled.

LPL activity in adipose, muscle, and heart, and plasma CETP activity were not different among the experimental groups at 9 mo (Table 9). The relative mRNA expression of liver MTTP was higher in TPN-IUGR pigs than that in the TPN-control group at 9 mo (Figure 4).

**TABLE 9:** LPL activity in adipose tissue, muscle, and heart, and plasma CETP activity in TPN-control, TPN-B+C, TPN-IUGR, and SowFed Yucatan miniature pigs at 9 mo

	TPN-control	TPN-B+C	TPN-IUGR	SowFed
LPL activity (mU⋅mg of protein^–1^)				
Subcutaneous fat	16.118 ± 6.474	14.221 ± 6.418	14.553 ± 5.858	12.81 ± 5.3512
Muscle	13.097 ± 3.255	11.195 ± 1.737	13.772 ± 4.641	13.620 ± 1.876
Heart	9.842 ± 3.038	9.525 ± 1.797	10.617 ± 1.378	9.549 ± 2.644
CETP activity (pmol⋅μL^–1^⋅h^–1^)	0.037 ± 0.008	0.035 ± 0.009	0.036 ± 0.010	0.036 ± 0.005

Values are means ± SD; *n* = 6–8.

Abbreviations: TPN, total parenteral nutrition; TPN-control, TPN-control diet; TPN-B+C, TPN-control diet supplemented with betaine and creatine; TPN-IUGR, IUGR piglets fed TPN-control diet; SowFed, suckled.

One-way analysis of variance with Dunnett’s post hoc test comparing with TPN-control: *P* > 0.05.

### Other biomarkers for cardiovascular disease

Cysteine and homocysteine levels were not different among the experimental groups at 9 mo in liver or plasma. Plasma DMG was higher in the TPN-IUGR pigs than in the TPN-control pigs at 9 mo (Table 10). At the end of TPN feeding, plasma cysteine concentrations were not different; however, plasma homocysteine levels were higher in the TPN-IUGR group than in the TPN-control group (Table 10). DMG levels were higher in TPN-B+C and SowFed pigs than TPN-control pigs at 21 d (Table 10).

**TABLE 10:** Plasma and liver methyl-related metabolites in TPN-control, TPN-B+C, TPN-IUGR, and SowFed Yucatan miniature piglets

	Age	TPN-control	TPN-B+C	TPN-IUGR	SowFed
Plasma					
Homocysteine (μmol⋅L^–1^)	21 d	23.8 ± 3.4	18.7 ± 4.8	46.2 ± 20.7^1^	25.6 ± 10.2
Cysteine (μmol⋅L^–1^)	21 d	258.1 ± 42.2	271.8 ± 50.4	239.3 ± 41.1	241.0 ± 23.2
Glutathione (μmol⋅L^–1^)	21 d	12.3 ± 6.0	8.0 ± 3.5	9.0 ± 3.5	8.6 ± 4.6
Dimethylglycine (μmol⋅L^–1^)	21 d	5.3 ± 2.3	18.6 ± 5.5^2^	4.4 ± 3.3	15.5 ± 3.3^2^
Betaine (μmol⋅L^–1^)	21 d	64.2 ± 16.8	358.4 ± 90.5^2^	35.2 ± 20.3	44.2 ± 11.4
Choline (μmol⋅L^–1^)	21 d	8.7 ± 1.7	11.8 ± 4.0	5.9 ± 2.2	8.0 ± 2.7
Homocysteine (μmol⋅L^–1^)	9 mo	35.2 ± 5.1	37.2 ± 7.1	34.6 ± 5.3	35.7 ± 4.9
Cysteine (μmol⋅L^–1^)	9 mo	331.2 ± 60.9	332.9 ± 42.3	335.9 ± 41.6	347.9 ± 31.3
Glutathione (μmol⋅L^–1^)	9 mo	7.4 ± 0.8	7.7 ± 1.7	7.1 ± 0.7	7.9 ± 1.3
Methionine (μmol⋅L^–1^)	9 mo	8.7 ± 2.6	7.7 ± 1.3	10.0 ± 3.5	9.4 ± 3.3
Dimethylglycine (μmol⋅L^–1^)	9 mo	1.40 ± 0.3	1.5 ± 0.3	2.3 ± 0.8^3^	1.5 ± 0.4
Betaine (μmol⋅L^–1^)	9 mo	105.8 ± 26.7	117.9 ± 43.6	114.4 ± 32.9	129.4 ± 44.5
Choline (μmol⋅L^–1^)	9 mo	6.4 ± 1.9	5.8 ± 1.8	6.9 ± 1.8	5.5 ± 1.2
Liver					
Homocysteine (μmol⋅g^−1^)	9 mo	0.097 ± 0.028	0.088 ± 0.019	0.105 ± 0.043	0.083 ± 0.028
Cysteine (μmol⋅g^−1^)	9 mo	0.096 ± 0.047	0.071 ± 0.030	0.068 ± 0.024	0.075 ± 0.024

Values are means ± SD; *n* = 7–8.

Abbreviations: TPN, total parenteral nutrition; TPN-control, TPN-control diet; TPN-B+C, TPN-control diet supplemented with betaine and creatine; TPN-IUGR, IUGR piglets fed with TPN-control diet; SowFed, suckled.

1 *P* < 0.001; one-way analysis of variance with Dunnett’s post hoc test comparing with TPN-control.

2 *P* < 0.0001; one-way analysis of variance with Dunnett’s post hoc test comparing with TPN-control.

3 *P* < 0.05; one-way analysis of variance with Dunnett’s post hoc test comparing with TPN-control.

## Discussion

TPN is a life-saving feeding method that has considerably increased infant survival rates since the early 1970s [4,56]. However, TPN feeding in the early neonatal period has been associated with changes in metabolism [6,7]. Studies have shown that TPN administration to neonates leads to acute dyslipidemia [57,58]; however, the persistence of altered lipid metabolism into adulthood remains unknown. We hypothesized that metabolic alterations due to early TPN feeding would persist into adulthood, highlighting the long-term effects of early TPN feeding. Supplementing TPN with betaine and creatine may negate this effect by enhancing the availability of methyl groups and preventing epigenetic changes that lead to a higher risk for dyslipidemia in adulthood. We expected to identify long-term effects of TPN feeding by comparing the TPN-control group to the SowFed group (clinical control) in adulthood. Comparing TPN-control with TPN-B+C group will indicate whether feeding betaine- and creatine-supplemented TPN in the neonatal period will prevent TPN-induced metabolism in adulthood. Finally, comparing TPN-control with TPN-IUGR will answer whether IUGR exacerbates TPN-induced outcomes. Our study is the first to show that neonatal TPN feeding leads to postprandial dyslipidemia in adulthood. Importantly, these pigs were fed the same grower diet from 3 wk of age to adulthood (9 mo) and dietary treatments only occurred for 2 wk as neonates; thus, postprandial dyslipidemia observations in adulthood were entirely due to nutritional stressors in early life. Moreover, as hypothesized, IUGR exacerbated TPN-induced postprandial dyslipidemia.

We demonstrated that feeding TPN to piglets early in life led to exaggerated postprandial triglyceridemia in adulthood compared with pigs that suckled as neonates. Postprandial dyslipidemia is strongly associated with endothelial dysfunction, and is thus a risk factor for atherosclerosis, CVD, and diabetes [59]. Therefore, our plasma and CM kinetics in adulthood indicate that compared with feeding sow milk in the early neonatal age, TPN feeding during early life leads to a reduced ability to clear postprandial dietary lipids, which may increase the risk of developing atherosclerosis, CVD, and diabetes in adulthood.

Mechanistically, feeding TPN in the neonatal period did not seem to affect dietary fat digestion or absorption in adulthood, considering there was no difference in time to peak plasma TG or CM TG between the TPN-fed pigs and sow-fed pigs in adulthood. Studies have shown that because TPN bypasses the gut, this route of feeding suppresses blood flow to the intestine and leads to intestinal villus atrophy, especially in the duodenum and jejunum, where fat absorption takes place [5,[60], [61], [62]]. However, this TPN-induced villus and crypt atrophy in the neonatal period did not seem to persist into adulthood, as indicated by the lack of differences in duodenal or proximal jejunum villus height and crypt depth between the TPN-fed pigs and the SowFed pigs (Table 7). Taken together, because gut atrophy did not persist into adulthood (Table 8), fat absorptive capacity was likely not altered, and time to reach peak plasma TG after an oral fat bolus dose was not affected by early feeding treatment.

Compared with SowFed pigs, higher postprandial peak TG concentrations in TPN-control pigs suggest their inability to clear TG-rich lipoproteins from circulation. The clearance of circulating CM in the postprandial state mainly depends on adipocyte and muscle LPL activities [63,64]. We measured adipose, muscle, and cardiac tissue LPL activity to evaluate the capacity of these tissues in the clearance of postprandial TG. However, LPL activity was not different in any measured tissue regardless of early diet (Table 9). Among these tissues, adipose is the greatest net importer of dietary fat in the postprandial stage [65]. The absence of increased LPL-specific activity in adipose tissue of the TPN-control group could explain the higher plasma TG levels postprandially in this group. However, we could not measure total LPL activity in the blood, as we did not inject heparin at necropsy, which would have compromised other parameters measured in the study.

It is unclear whether postprandial TG responses determine the fasting TG levels or vice versa. We observed higher postprandial TG in TPN-control pigs in adulthood but no differences in the fasted plasma TG. This discrepancy suggests that the development of higher postprandial TG levels due to lack of clearance ability might be an earlier marker of dyslipidemia than fasting TG concentration. If the magnitude of the postprandial TG continues to remain high, the fasting TG concentrations may also eventually stabilize at higher levels. The addition of betaine and creatine to neonatal TPN did not affect growth performance or lipid metabolism in later life. The ability to clear postprandial plasma TG and CM TG after consuming a fat bolus in TPN-B+C pigs also did not differ from that of TPN-control pigs. With respect to other metabolic syndrome biomarkers, the nonsignificant fasting plasma glucose and insulin concentrations suggest that our pigs may have been too young to exhibit any fasting biomarkers of metabolic syndrome.

IUGR neonates make up ∼10%–15% [66] of all neonates worldwide, and they often receive TPN in early life. Previous studies have shown that sow-fed IUGR piglets experience accelerated growth (i.e., catch-up growth) [14,15], greater adiposity, and impaired lipid metabolism as young adults [15]. Thus, comparing TPN-IUGR with TPN-control pigs will resolve whether being IUGR will exacerbate the deleterious effects of feeding TPN in early life on lipid metabolism in adulthood. We found that IUGR piglets had caught up in body weight by 1 mo, after TPN feeding. Catch-up growth was similar to our previous study when sow milk replacer formula was used in IUGR piglets [14]. The increased body growth rate of TPN-IUGR pigs compared with the TPN-control group during the 1–4 and 4–6 mo is likely due to the development of obesity into adolescence, as seen previously in formula-fed IUGR pigs [14]. Thus, our data confirm that IUGR pigs demonstrate catch-up growth around weaning age to the end of presexual maturity age (4 mo), regardless of the mode of feeding. This catch-up growth was reduced in postsexual maturity, and body weights paralleled non-IUGR pigs in the postsexual maturity phase (6–8 mo). However, TPN-IUGR pigs were obese as adults, as indicated by increased backfat thickness and plasma NEFA levels compared with the TPN-control group. Because we did not measure individual feed intakes (the feed intakes in Table 3 are estimates from 2% group feeding), we could not conclude whether this catch-up growth was due to higher feed efficiency, or higher feed intake.

The most interesting finding of the current study is that although early TPN feeding is a necessary life-saving measure in IUGR neonates, they are particularly sensitive to the long-term metabolic consequences of TPN, predisposing them to metabolic disorders such as dyslipidemia in adulthood. This conclusion was supported by the worsened fasting lipoprotein profiles and TG and TC levels in the lipoprotein subfractions in TPN-fed IUGR pigs. Increased obesity in TPN-IUGR pigs, as indicated by more subcutaneous fat, may have led to higher fasting NEFA levels in the circulation, which then enhanced the flux of free fatty acids into the liver, causing higher TG in the liver compared with TPN-control pigs. The higher hepatic influx of free fatty acids may have led to greater VLDL secretion, as indicated by higher MTTP gene expression, causing higher fasting non-HDL-cholesterol levels and TG in VLDL subfractions. Although VLDL particles of TPN-IUGR pigs had greater TG content and reduced cholesterol levels compared with TPN-control pigs, plasma CETP activity, which facilitates the transport of cholesterol esters and TG between lipoproteins, was not different between the groups (Table 9).

The elevated levels of postprandial plasma TG in the TPN-IUGR pigs in adulthood are likely due to elevated VLDL levels (Figure 3), as plasma TG clearance rate was significantly slower. However, the CM TG clearance rate was not different. This conclusion is supported by the higher liver MTTP expression levels and the positive correlation between plasma TG clearance rate and TG levels in the VLDL subfraction. Other studies have also shown that the majority of the increment of postprandial lipoprotein in the clearance phase is due to the presence of VLDL remnants, not CM remnants, based on the apo100/apo48 ratio and the particle size, which fluctuates over 6 h after a fat bolus intake [67]. The slower VLDL clearance in TPN-IUGR pigs could be due to low LPL activity or low VLDL receptor in the extrahepatic tissues such as adipocytes, muscle, and brain, as the majority of the VLDL remnants are removed from the plasma by the VLDL receptor in peripheral tissues after removing TG from VLDL particles by LPL.

Previous studies showed that IUGR pigs have lower methionine cycle enzyme activity, including 30% lower betaine:homocysteine methyltransferase (BHMT) activity and 20% lower cystathionine gamma lyase (CGL) activity [27,68]. These lower enzyme capacities limit the disposal of homocysteine, which may explain the higher homocysteine levels in TPN-IUGR piglets in the TPN phase (21 d). However, adult pigs (9 mo) did not show hyperhomocysteinemia, suggesting that reduced BHMT and CGL activity in IUGR pigs may not persist into adulthood or homocysteinemia was maintained via increased renal excretion in adulthood.

When the TPN diet was supplemented with betaine (TPN-B+C), plasma levels of DMG, the product of BHMT activity and a nonconventional biomarker for CVD [69], were higher than TPN-control in the TPN phase (21 d), but also were within the same range as the clinical reference group (SowFed). These data suggest that increased DMG levels in the B+C supplemented group in the piglet stage are due to supplemented betaine to TPN, and the resulting DMG levels represent normal levels. However, increased DMG levels in the IUGR pigs at adulthood suggest that, although hyperhomocysteinemia did not persist into adulthood, TPN-IUGR pigs may still be at risk for developing CVD in adulthood.

Taken together, our data provide evidence that feeding TPN in the neonatal period permanently altered the lipid metabolism of pigs, predisposing them to increased risk of CVD, and these deleterious effects persist into young adulthood. Supplementing betaine and creatine to neonatal TPN did not improve lipid metabolism in adult pigs, compared with pigs fed control TPN as neonates. Although it is common that IUGR neonates receive TPN as a life-saving measure early in life, this feeding regimen permanently programs poor lipid metabolism, predisposing them to develop obesity and dyslipidemia in adulthood. Understanding the effects of feeding TPN to IUGR neonates and finding novel nutrition interventions that reduce chronic disease risk has important implications in infant nutrition. Although we have previously shown that IUGR independently affects postprandial dyslipidemia in adulthood [15], in this study, the TPN-IUGR treatment also showed postprandial dyslipidemia in later life. However, whether IUGR is more important than TPN is unclear. The findings of this study will help establish phenotypic outcomes in adulthood as a result of feeding TPN in early life and will shed light on understanding the mechanisms of early programming. Thus, further novel nutritional interventions can be designed to offset the disease risk in adulthood.

## Author contributions

The authors’ responsibilities were as follows – RFB, JAB: designed the research; RSR, RFB: wrote the article and have primary responsibility for final content; L-AH: conducted the initial animal work; KA: assisted in animal experiments; EWR: provided the technical support analyzing samples used in LCMS; RSR: conducted the research and analyzed the data; and all authors; have read and approved the final manuscript.

## Funding

This project was supported by the Canadian Institutes of Health Research.

## Conflict of interest

RFB is an Editorial Board Member for the Journal of Nutrition and played no role in the Journal’s evaluation of the manuscript. All other authors report no conflicts of interest.

## References

[1] Bovolini A., Garcia J., Andrade M.A., Duarte J.A. (2021). Metabolic syndrome pathophysiology and predisposing factors. Int. J. Sports Med..

[2] Hoffman D.J., Reynolds R.M., Hardy DB. (2017). Developmental origins of health and disease: current knowledge and potential mechanisms. Nutr. Rev.

[3] Calkins K.L., Venick R.S., Devaskar S.U. (2014). Complications associated with parenteral nutrition in the neonate. Clin. Perinatol..

[4] Vinnars E., Wilmore D. (2003). History of parenteral nutrition. J. Parenter. Enteral Nutr..

[5] Bertolo R.F.P., Pencharz P.B., Ball R.O. (1999). A comparison of parenteral and enteral feeding in neonatal piglets, including an assessment of the utilization of a glutamine-rich, pediatric elemental diet. J. Parenter. Enteral Nutr..

[6] Stoll B., Puiman P.J., Cui L., Chang X., Benight N.M., Bauchart-Thevret C. (2012). Continuous parenteral and enteral nutrition induces metabolic dysfunction in neonatal pigs. JPEN J. Parenter. Enter. Nutr..

[7] Stoll B., Horst D.A., Cui L., Chang X., Ellis K.J., Hadsell D.L. (2010). Chronic parenteral nutrition induces hepatic inflammation, steatosis, and insulin resistance in neonatal pigs. J. Nutr..

[8] Lavoie J.C., Bélanger S., Spalinger M., Chessex P. (1997). Admixture of a multivitamin preparation to parenteral nutrition: the major contributor to in vitro generation of peroxides. Pediatrics.

[9] Lucchinetti E., Lou P.H., Wawrzyniak P., Wawrzyniak M., Scharl M., Holtzhauer G.A. (2021). Novel strategies to prevent total parenteral nutrition-induced gut and liver inflammation, and adverse metabolic outcomes. Mol. Nutr. Food Res..

[10] Lim S., Kim J.W., Targher G. (2021). Links between metabolic syndrome and metabolic dysfunction-associated fatty liver disease. Trends Endocrinol. Metab..

[11] Rector R.S., Thyfault J.P., Wei Y., Ibdah J.A. (2008). Non-alcoholic fatty liver disease and the metabolic syndrome: an update. World J. Gastroenterol..

[12] Huxley R., Owen C.G., Whincup P.H., Cook D.G., Rich-Edwards J., Smith G.D. (2007). Is birth weight a risk factor for ischemic heart disease in later life?. Am. J. Clin. Nutr..

[13] Oken E., Gillman M.W. (2003). Fetal origins of obesity. Obes. Res..

[14] McKnight L.L., Myrie S.B., MacKay D.S., Brunton J.A., Bertolo R.F. (2012). Glucose tolerance is affected by visceral adiposity and sex, but not birth weight, in Yucatan miniature pigs. Appl. Physiol. Nutr. Metab..

[15] Myrie S.B., McKnight L.L., King J.C., McGuire J.J., Van Vliet B.N., Cheema S.K. (2017). Intrauterine growth-restricted Yucatan miniature pigs experience early catch-up growth, leading to greater adiposity and impaired lipid metabolism as young adults. Appl. Physiol. Nutr. Metab..

[16] Kerkhof G.F., Willemsen R.H., Leunissen R.W.J., Breukhoven P.E., Hokken-Koelega A.C.S. (2012). Health profile of young adults born preterm: negative effects of rapid weight gain in early life. J. Clin. Endocrinol. Metab..

[17] Singhal A. (2017). Long-term adverse effects of early growth acceleration or catch-up growth. Ann. Nutr. Metab..

[18] Bolisetty S., Osborn D., Schindler T., Sinn J., Deshpande G., Wong C.S. (2020). Standardised neonatal parenteral nutrition formulations – Australasian neonatal parenteral nutrition consensus update 2017. BMC Pediatr.

[19] Elefson S.K., Stoll B., Davis T.A., Fiorotto M.L., El-Kadi S.W., Genovese K. (2024). Adverse metabolic phenotypes in parenterally fed neonatal pigs do not persist into adolescence. J. Nutr..

[20] Randunu R.S., Bertolo R.F. (2020). The effects of maternal and postnatal dietary methyl nutrients on epigenetic changes that lead to non-communicable diseases in adulthood. Int. J. Mol. Sci..

[21] Waterland R.A., Jirtle R.L. (2004). Early nutrition, epigenetic changes at transposons and imprinted genes, and enhanced susceptibility to adult chronic diseases. Nutrition.

[22] Cai D., Jia Y., Lu J., Yuan M., Sui S., Song H. (2014). Maternal dietary betaine supplementation modifies hepatic expression of cholesterol metabolic genes via epigenetic mechanisms in newborn piglets. Br. J. Nutr..

[23] Waterland R.A., Dolinoy D.C., Lin J.R., Smith C.A., Shi X., Tahiliani K.G. (2006). Maternal methyl supplements increase offspring DNA methylation at Axin fused. Genesis.

[24] Burdge G.C., Lillycrop K.A., Phillips E.S., Slater-Jefferies J.L., Jackson A.A., Hanson M.A. (2009). Folic acid supplementation during the juvenile-pubertal period in rats modifies the phenotype and epigenotype induced by prenatal nutrition. J. Nutr..

[25] Elango R. (2020). Methionine nutrition and metabolism: insights from animal studies to inform human nutrition. J. Nutr..

[26] Riedijk M.A., Stoll B., Chacko S., Schierbeek H., Sunehag A.L., van Goudoever J.B. (2007). Methionine transmethylation and transsulfuration in the piglet gastrointestinal tract. Proc. Natl Acad. Sci. U S A..

[27] MacKay D.S., Brophy J.D., McBreairty L.E., McGowan R.A., Bertolo R.F. (2012). Intrauterine growth restriction leads to changes in sulfur amino acid metabolism, but not global DNA methylation, in Yucatan miniature piglets. J. Nutr. Biochem..

[28] Shoveller A.K., House J.D., Brunton J.A., Pencharz P.B., Ball R.O. (2004). The balance of dietary sulfur amino acids and the route of feeding affect plasma homocysteine concentrations in neonatal piglets. J. Nutr..

[29] Bauchart-Thevret C., Stoll B., Burrin D.G. (2009). Intestinal metabolism of sulfur amino acids. Nutr. Res. Rev..

[30] Brosnan J.T., Wijekoon E.P., Warford-Woolgar L., Trottier N.L., Brosnan M.E., Brunton J.A. (2009). Creatine synthesis is a major metabolic process in neonatal piglets and has important implications for amino acid metabolism and methyl balance. J. Nutr..

[31] Bertolo R.F., McBreairty L.E. (2013). The nutritional burden of methylation reactions. Curr. Opin. Clin. Nutr. Metab. Care..

[32] McBreairty L.E., McGowan R.A., Brunton J.A., Bertolo RF. (2013). Partitioning of [methyl-3H]methionine to methylated products and protein is altered during high methyl demand conditions in young Yucatan miniature pigs. J. Nutr..

[33] Deminice R., da Silva R.P., Lamarre S.G., Kelly K.B., Jacobs R.L., Brosnan M.E. (2015). Betaine supplementation prevents fatty liver induced by a high-fat diet: effects on one-carbon metabolism. Amino Acids.

[34] Myrie S.B., MacKay D.S., Van Vliet B.N., Bertolo R.F. (2012). Early programming of adult blood pressure in the low birth weight Yucatan miniature pig is exacerbated by a post-weaning high-salt-fat-sugar diet. Br. J. Nutr..

[35] Beigh S.H., Jain S. (2012). Prevalence of metabolic syndrome and gender differences. Bioinformation.

[36] Hegarty P.V., Allen C.E. (1978). Effect of pre-natal runting on the post-natal development of skeletal muscles in swine and rats. J. Anim. Sci..

[37] Dodge M.E., Bertolo R.F., Brunton J.A. (2012). Enteral feeding induces early intestinal adaptation in a parenterally fed neonatal piglet model of short bowel syndrome. JPEN J. Parenter. Enter. Nutr..

[38] Myrie S.B., McKnight L.L., Van Vliet B.N., Bertolo R.F. (2011). Low birth weight is associated with reduced nephron number and increased blood pressure in adulthood in a novel spontaneous intrauterine growth-restricted model in Yucatan miniature swine. Neonatology.

[39] Shoveller A.K., Brunton J.A., House J.D., Pencharz P.B., Ball R.O. (2003). Dietary cysteine reduces the methionine requirement by an equal proportion in both parenterally and enterally fed piglets. J. Nutr..

[40] Hurley W.L., Farmer C. (2015). The Gestating and Lactating Sow.

[41] Kolovou G.D., Mikhailidis D.P., Kovar J., Lairon D., Nordestgaard B.G., Ooi T.C. (2011). Assessment and clinical relevance of non-fasting and postprandial triglycerides: an expert panel statement. Curr. Vasc. Pharmacol..

[42] McAteer M.A., Grimsditch D.C., Vidgeon-Hart M., Benson G.M., Salter A.M. (2003). Dietary cholesterol reduces lipoprotein lipase activity in the atherosclerosis-susceptible Bio F (1)B hamster. Br. J. Nutr..

[43] Salter A.M., Mangiapane E.H., Bennett A.J., Bruce J.S., Billett M.A., Anderton K.L. (1998). The effect of different dietary fatty acids on lipoprotein metabolism: concentration-dependent effects of diets enriched in oleic, myristic, palmitic and stearic acids. Br. J. Nutr..

[44] Folch J., Lees M., Stanley G.H. (1957). A simple method for the isolation and purification of total lipids from animal tissues. J. Biol. Chem..

[45] Holm P.I., Ueland P.M., Kvalheim G., Lien E.A. (2003). Determination of choline, betaine, and dimethylglycine in plasma by a high-throughput method based on normal-phase chromatography-tandem mass spectrometry. Clin. Chem..

[46] Kirsch S.H., Herrmann W., Rabagny Y., Obeid R. (2010). Quantification of acetylcholine, choline, betaine, and dimethylglycine in human plasma and urine using stable-isotope dilution ultra performance liquid chromatography-tandem mass spectrometry. J. Chromatogr. B Analyt. Technol. Biomed. Life Sci..

[47] Vester B., Rasmussen K. (1991). High performance liquid chromatography method for rapid and accurate determination of homocysteine in plasma and serum. Eur. J. Clin. Chem. Clin. Biochem..

[48] Thillayampalam K. (2015).

[49] Bidlingmeyer B.A., Cohen S.A., Tarvin T.L. (1984). Rapid analysis of amino acids using pre-column derivatization. J. Chromatogr..

[50] Bartlett G.R. (1959). Phosphorus assay in column chromatography. J. Biol. Chem..

[51] Chomczynski P., Sacchi N. (2006). The single-step method of RNA isolation by acid guanidinium thiocyanate-phenol-chloroform extraction: twenty-something years on. Nat. Protoc..

[52] Chen Y.J., Chen C.C., Li T.K., Wang P.H., Liu L.R., Chang F.Y. (2012). Docosahexaenoic acid suppresses the expression of FoxO and its target genes. J. Nutr. Biochem..

[53] Nygard A.B., Jørgensen C.B., Cirera S., Fredholm M. (2007). Selection of reference genes for gene expression studies in pig tissues using SYBR green qPCR. BMC Mol. Biol..

[54] Park S.J., Kwon S.G., Hwang J.H., Park D.H., Kim T.W., Kim C.W. (2015). Selection of appropriate reference genes for RT-qPCR analysis in Berkshire, Duroc, Landrace, and Yorkshire pigs. Gene.

[55] Vandesompele J., De Preter K., Pattyn F., Poppe B., van Roy N., de Paepe A. (2002). Accurate normalization of real-time quantitative RT-PCR data by geometric averaging of multiple internal control genes. Genome Biol.

[56] Puntis J.W. (2006). Nutritional support in the premature newborn. Postgrad. Med. J..

[57] Jain A.K., Stoll B., Burrin D.G., Holst J.J., Moore D.D. (2012). Enteral bile acid treatment improves parenteral nutrition-related liver disease and intestinal mucosal atrophy in neonatal pigs. Am. J. Physiol. Gastrointest. Liver Physiol..

[58] Nghiem-Rao T.H., Dahlgren A.F., Kalluri D., Cao Y., Simpson P.M., Patel S.B. (2016). Influence of gestational age and birth weight in neonatal cholesterol response to total parenteral nutrition. J. Clin. Lipidol..

[59] Ansar S., Koska J., Reaven P.D. (2011). Postprandial hyperlipidemia, endothelial dysfunction and cardiovascular risk: focus on incretins. Cardiovasc. Diabetol..

[60] Niinikoski H., Stoll B., Guan X., Kansagra K., Lambert B.D., Stephens J. (2004). Onset of small intestinal atrophy is associated with reduced intestinal blood flow in TPN-fed neonatal piglets. J. Nutr..

[61] Shaw D., Gohil K., Basson M.D. (2012). Intestinal mucosal atrophy and adaptation. World J. Gastroenterol..

[62] Watts G.F., Playford D.A. (1998). Dyslipoproteinaemia and hyperoxidative stress in the pathogenesis of endothelial dysfunction in non-insulin dependent diabetes mellitus: an hypothesis. Atherosclerosis.

[63] Paglialunga S., Cianflone K. (2007). Regulation of postprandial lipemia: an update on current trends. Appl. Physiol. Nutr. Metab..

[64] Fielding B. (2011). Tracing the fate of dietary fatty acids: metabolic studies of postprandial lipaemia in human subjects. Proc. Nutr. Soc..

[65] Suhag A., Berghella V. (2013). Intrauterine growth restriction (IUGR): etiology and diagnosis. Curr. Obstet. Gynecol Rep..

[66] Nakajima K., Nakano T., Tokita Y., Nagamine T., Inazu A., Kobayashi J. (2011). Postprandial lipoprotein metabolism: VLDL vs chylomicrons. Clin. Chim. Acta..

[67] McBreairty L.E., Robinson J.L., Furlong K.R., Brunton J.A., Bertolo R.F. (2015). Guanidinoacetate is more effective than creatine at enhancing tissue creatine stores while consequently limiting methionine availability in Yucatan miniature pigs. PLOS ONE.

[68] Svingen G.F., Schartum-Hansen H., Ueland P.M., Pedersen E.R., Seifert R., Ebbing M. (2015). Elevated plasma dimethylglycine is a risk marker of mortality in patients with coronary heart disease. Eur. J. Prev. Cardiol..

